# Supplementary material for: Biogeography, diversity and environmental relationships of shelf and deep-sea benthic Amphipoda around Iceland
Source: PeerJ. 2021 Aug 11;9:e11898. doi: 10.7717/peerj.11898 (PMC8364320; doi:10.7717/peerj.11898)
Supplement: Supplemental Information 1 [file peerj-09-11898-s001.docx]

**Appendix 1:** Descriptive statistics for environmental parameters, four environmental clusters and all hexagonal cells.

|  | **Coastal (N=88)** | **GFIR (N=101)** | **Deep North (N=140)** | **Deep South (N=140)** | **All (N=469)** |
| --- | --- | --- | --- | --- | --- |
| **Depth (m)** |  |  |  |  |  |
| Mean (SD) | -345 (278) | -839 (446) | -2230 (900) | -2400 (489) | -1630 (1050) |
| Median [Min, Max] | -275 [-1140, -11.0] | -750 [-2030, -149] | -2080 [-3730, -613] | -2400 [-3330, -1320] | -1570 [-3730, -11.0] |
| **Dissolved Oxygen (µmol/m²)** |  |  |  |  |  |
| Mean (SD) | 306 (20.0) | 266 (19.8) | 298 (1.50) | 267 (5.11) | 284 (21.7) |
| Median [Min, Max] | 300 [271, 366] | 266 [228, 315] | 298 [295, 301] | 268 [253, 289] | 284 [228, 366] |
| **Dissolved Iron (µmol/m²)** |  |  |  |  |  |
| Mean (SD) | 0.00128 (0.000457) | 0.000848 (0.000133) | 0.000727 (0.0000830) | 0.000704 (0.0000271) | 0.000850 (0.000301) |
| Median [Min, Max] | 0.00120 [0.000662, 0.00256] | 0.000793 [0.000674, 0.00125] | 0.000683 [0.000647, 0.000960] | 0.000696 [0.000652, 0.000783] | 0.000744 [0.000647, 0.00256] |
| **pH** |  |  |  |  |  |
| Mean (SD) | 8.20 (0.0180) | 8.18 (0.0119) | 8.20 (0.0154) | 8.18 (0.0270) | 8.19 (0.0215) |
| Median [Min, Max] | 8.20 [8.15, 8.26] | 8.18 [8.15, 8.20] | 8.20 [8.16, 8.23] | 8.18 [8.15, 8.26] | 8.19 [8.15, 8.26] |
| **Phytobiomass (kg/m²)** |  |  |  |  |  |
| Mean (SD) | 1.92 (2.10) | 0.365 (0.390) | 0.0221 (0.00719) | 0.0333 (0.0725) | 0.456 (1.17) |
| Median [Min, Max] | 1.41 [0.0320, 10.1] | 0.221 [0.0233, 1.59] | 0.0200 [0.0196, 0.0680] | 0.0211 [0.0200, 0.776] | 0.0263 [0.0196, 10.1] |
| **Temperature (°C)** |  |  |  |  |  |
| Mean (SD) | 5.62 (3.51) | 6.27 (1.66) | -0.652 (0.472) | 3.28 (0.654) | 3.19 (3.26) |
| Median [Min, Max] | 5.58 [0.111, 12.0] | 6.19 [2.88, 10.4] | -0.855 [-1.04, 1.05] | 3.03 [2.32, 4.79] | 3.00 [-1.04, 12.0] |
