# Supplementary figures and images for: Biogeography, diversity and environmental relationships of shelf and deep-sea benthic Amphipoda around Iceland
Source: PeerJ. 2021 Aug 11;9:e11898. doi: 10.7717/peerj.11898 (PMC8364320; doi:10.7717/peerj.11898)

+

-

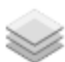

## Cluster

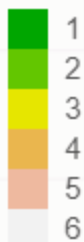

Island

Cluster

300 km

200 mi

United Kingdom

Supplement: Supplemental Information 4 [file peerj-09-11898-s004.pdf]
